# Molecular and cytogenetic data for *Rhamphichthys* Müller and Troschel, 1848 (Rhamphichthyidae - Gymnotiformes): Evidence of chromosomal differentiation in *R. heleios*, *R. hahni*, *R. rostratus* and *R. pantherinus*

**DOI:** 10.1590/1678-4685-GMB-2025-0092

**Published:** 2026-08-03

**Authors:** Marlyson Jeremias Rodrigues da Costa, Patricia Correa da Silva, Cleusa Yoshiko Nagamachi, Wilsea Maria B. Figueiredo Ready, Jonathan Stuart Ready, Julio Cesar Pieczarka

**Affiliations:** 1 Universidade Federal do Pará, Instituto de Ciências Biológicas, Centro de Estudos Avançados da Biodiversidade, Belém, PA, Brazil

**Keywords:** Cytogenetics, B chromosome, Amazon biodiversity, DNA barcoding

## Abstract

*Rhamphichthys* (Gymnotiformes), a genus with uncertain taxonomy due to rarity and morphological similarity, exhibits greater diversity in the Amazon Basin. Phylogenetic analysis of COI data from specimens collected in the Amazon Basin, in Guamá River (Belém and Barcarena), Caripetuba River, and Anequara River (Abaetetuba) and deposited in the collection of the Museu Paraense Emílio Goeldi identified two major monophyletic clades within *Rhamphichthys*, encompassing *R. pantherinus* and *R. rostratus*. Cytogenetic analysis of 24 specimens, both with 2n=50 but differing fundamental number (FN) and karyotype formula (KF), enabled their differentiation. Although 2n=50 is likely ancestral, karyotype constitution varies among species. By combining cytogenetic and molecular data, we reclassified the previously described *Rhamphichthys* “*marmoratus*” (2n=50, FN=94, KF=44m/sm+6st/a) as *R. heleios*, and previous *R. rostratus* (2n=50, FN=92, KF=42m/sm+8st/a) as *R. pantherinus*. This study provides the first cytogenetic data for the real *R. rostratus* species and reports novel B chromosomes within the Rhamphichthyidae family.

## Introduction

The genus *Rhamphichthys* Müller And Troschel, 1848 stands out as the most species-rich genus of the family Rhamphichthyidae (Gymnotiformes), currently including nine valid species: *Rhamphichthys rostratus* Linnaeus, 1766; *Rhamphichthys lineatus* Castelnau, 1855; *Rhamphichthys pantherinus* Castelnau, 1855; *Rhamphichthys hahni* Minken, 1937; *Rhamphichthys apurensis* Fernández-Yépez, 1968; *Rhamphichthys atlanticus* Triques, 1999; *Rhamphichthys drepanium* Triques, 1999; *Rhamphichthys longior* Triques, 1999; *Rhamphichthys heleios* Carvalho and Albert, 2015. In a recent publication, Carvalho and Albert (2023) consider the species *Rhamphichthys apurensis*, *R. drepanium*, *R. hahni*, *R. heleios*, *R. lineatus*, *R. pantherinus*, and *R. rostratus* to be valid. In turn, *R. atlanticus* and *R. longior* are proposed as junior synonyms of *R. pantherinus*.

The taxonomic history of this genus includes various rearrangements with previous species allocations in other gymnotiform genera, synonymizations, and descriptions of new species (Triques, 1994, 1999; Carvalho and Albert, 2015). In addition, these species have great morphological similarities, color patterns, and habitat use (Reis et al., 2003). It is therefore possible that the genus has an underestimated species richness, especially as these fish also have a low sample density in scientific collections (Alves-Gomes, 1998; Albert and Crampton, 2003; Crampton and Albert, 2005; Crampton, 2011). 

Despite this context, few studies using alternative methods to morphology have been employed for this genus. Evidence of this lack of additional data is that only three species were analyzed cytogenetically (Mendes et al., 2012; Silva et al., 2013) and few studies present a consistent resolution for species using molecular markers (Carvalho, 2013; Tagliacollo et al., 2016) providing uncertainties regarding the taxonomic status of many lineages, even more so if we consider that species commonly occur sympatrically in the Amazon basin. 

With the proposal to provide the identification of species in a faster, more accurate, and automated way, the Cytochrome C Oxidase I (COI) gene as a DNA barcode has been widely used, mainly in supporting animal taxonomy, understanding biogeographical patterns and helping conservation biology (Hebert et al., 2003; Hebert and Gregory, 2005; Hobern, 2021). The efficiency of DNA barcoding is evident when the intraspecific diversity of the COI gene is lower than the interspecific diversity. From this, Puillandre et al. (2012) proposed the Automatic Barcode Gap Discovery (ABGD) species delimitation analysis method. ABGD uses a clustering algorithm to group DNA sequences automatically, considering genetic differences and distance gaps. This allows the method to be used to identify similar but genetically distinct species, even when no genetic references are available.

Considering the context that the genus *Rhamphichthys* needs information regarding complementary markers to morphology, the present work compiled molecular (COI) and cytogenetic (diploid number - 2n, karyotypic formula - KF and chromosomal banding) data for a better understanding of the diversity present in the genus. We present new cytogenetic data for the genus and aim to confirm karyotypic associations to species names considering the systematic changes that have occurred in the genus.

## Material and Methods

### Ethics statement

The Animal Ethics Committee (Comitê de Ética Animal) from Universidade Federal do Pará (UFPA), authorized the present study (Permit 68-2015). JCP has a permanent field permit, number 13248 from “Instituto Chico Mendes de Conservação da Biodiversidade”. The Cytogenetics Laboratory from UFPA has a special permit number 19/2003 from the Ministry of Environment for sample transport and 52/2003 for using the samples for research. 

### Samples and chromosomal preparation

*Rhamphichthys* were collected in the Amazon Basin, in the Guamá (Belém), Arienga (Barcarena), Caripetuba, and Anequara (Abaetetuba) rivers (21 *R. rostratus* and 3 *R. pantherinus*), and their representatives were deposited in the collection of the Museu Paraense Emílio Goeldi (MPEG) (Table 1). Samples were morphologically identified using measures according to what was proposed by Carvalho et al. (2011) and Carvalho and Albert (2011) where a clear distinction between the species was made based on the measures of snout length relative to head length (Table S1).

**Table 1 -:** New samples analyzed in this work. LA = Lower Amazon.

Locality	Samples	Species	Karyotype	B chromosomes
Anequara River, Abaetetuba 01°40’42.6”S, 049°00’16.6”W	P1180 (Female)	*R. rostratus* (LA)	48m/sm+2st/a	8
	P1181 (Female)	*R. rostratus* (LA)	48m/sm+2st/a	8
	P1187 (Male)	*R. rostratus* (LA)	48m/sm+2st/a	7-8
	P1188 (Female)	*R. rostratus* (LA)	48m/sm+2st/a	8
Caripetuba River Abaetetuba 01°37’23,49”S 048°55’33”W	P1285 (Male)	*R. pantherinus*	42m/sm+8st/a	0
	P1286 (Female)	*R. pantherinus*	42m/sm+8st/a	0
	P1899 (Male)	*R. pantherinus*	42m/sm+8st/a	0
	P1294 (Male)	*R. rostratus* (LA)	48m/sm+2st/a	5-8
	P1900 (Male)	*R. rostratus* (LA)	48m/sm+2st/a	7-8
	P2749 (Male)	*R. rostratus* (LA)	48m/sm+2st/a	8
	P2757 (Female)	*R. rostratus* (LA)	48m/sm+2st/a	7-8
	P2758 (Female)	*R. rostratus* (LA)	48m/sm+2st/a	8
	P2759 (Male)	*R. rostratus* (LA)	48m/sm+2st/a	8
	P2764 (Male)	*R. rostratus* (LA)	48m/sm+2st/a	8
	P3070 (Male)	*R. rostratus* (LA)	48m/sm+2st/a	8
Guamá River, Belém 01°28’33.88”S 048°27’08.73”W	P1164 (Female)	*R. rostratus* (LA)	48m/sm+2st/a	6
	P1165 (Male)	*R. rostratus* (LA)	48m/sm+2st/a	6
	P1190 (Male)	*R. rostratus* (LA)	48m/sm+2st/a	5-6
Arienga River, Barcarena 1º34’25.36”S 48º45’55.21” W	P1541 (Female)	*R. rostratus* (LA)	48m/sm+2st/a	8-10
	P1543 (Female)	*R. rostratus* (LA)	48m/sm+2st/a	6-10
	P1544 (Female)	*R. rostratus* (LA)	48m/sm+2st/a	9-10
	P1545 (Male)	*R. rostratus* (LA)	48m/sm+2st/a	10
	P1546 (Male)	*R. rostratus* (LA)	48m/sm+2st/a	9-10
	P1547 (Female)	*R. rostratus* (LA)	48m/sm+2st/a	10

*Molecular analysis*

**Figure 1 -:**
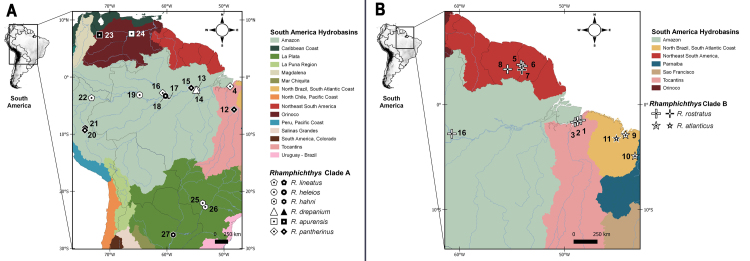
Location map of molecular samples (filled symbol) and type locality (empty symbol) of *Rhamphichthys* species. The hydrographic network is defined by the blue lines and colors represented by the South American Hydrobasins. A) *Rhamphichthys lineatus* - 21, 22 (Carvalho and Albert, 2015); *R. heleios* - 13, 18, 19; (Silva et al., 2013; Janzen et al., 2022); *R. hahni* - 25-27 (Pereira et al., 2013) *R. drepanium* - 14, 17 (Janzen *et al*., 2022); *R. apurensis* - 23, 24 (Tagliacollo et al., 2016); *R. pantherinus* - 4, 12 *type locality *R. marmoratus*, 15 *type locality *R. longior*, 16, 20 *type locality *R. pantherinus* (Maldonado-Ocampo et al., 2013; present study); B) *R. rostratus* - 1-3, 5-8, 16 (Maldonado-Ocampo *et al*., 2013; Tagliacollo *et al*., 2016; Papa et al., 2021; present study); *R. atlanticus* - 9-11 (Birindelli, JL - BOLD:ADC5116). This map was made using the free software Quantum-Gis version 2.10.1. The databases were obtained from DIVA-GIS. The files provided by DIVA-GIS are free of charge.

Previous tissue samples of *R. “marmoratus”* (*R. pantherinus*) from Mamirauá, Amazonas state, and *R. rostratus* from the Rio Parú, Pará state, with existing karyotypic descriptions (Silva et al., 2013) were also included for sequence based analyses. One specimen of *Rhamphichthys heleios* (1316), two of *R. pantherinus* (1899 and 1900), and six of *R. rostratus* (1164, 1165, 1187, 1188, 1544, and 2750) had genomic DNA extracted using the chloroform-phenol method (Sambrook and Russell, 2006). Amplification of a fragment of the mitochondrial gene COI was performed by the PCR technique using primers LIICO1F (GATTTTTCTCAACTAACCAYAAAGA) and LIICO1R (ACTTCTGGGTGTCCGAARAAYCARAA) (Cardoso et al., 2018). After amplification, the product was purified and sequenced using an ABI 3130-Genetic Analyzer (Applied Biosystems) and the manufacturer’s BigDye kit. Pre-existing sequences for seven species of the genus *Rhamphichthys* were obtained from GenBank and BOLD as well the outgroup taxa *Gymnor hamphichthys britskii* Carvalho, Ramos and Albert, 2011, *G. hypostomus* Géry and Vu, 1964*, G. rondoni* Miranda Ribeiro, 1920, *Steatogenys elegans* Steindachner, 1880, *S. duidae* La Monte, 1929 and *Hypopygus lepturus* Hoedeman, 1962. All sequences used are listed in Table S2. The maps in Figure 1 show the locations from each sequence of *Rhamphichthys* used here.

All sequences were aligned with the software MEGA 11 (Tamura et al., 2021) using the MUSCLE algorithm (Edgar, 2004) and the values of intra and interspecific genetic distances were calculated, using the Kimura-2-Parameters (K2P - Table S3) substitution model (Kimura, 1980). The best evolutionary model and phylogenetic reconstructions were performed using the ModelFinder (Kalyaanamoorthy et al., 2017) implemented in the IQ-Tree web server (http://iqtree.cibiv. univie.ac.at/) using 48 sequences with 678 nucleotide sites with Bayesian selection criteria. For reconstruction of Maximum Likelihood (ML) trees, tree reconstruction + ultrafast bootstrap estimator with 1000 replicates and TVM+F+I+G4 model in IQ-Tree web server (Nguyen et al., 2015; Hoang et al., 2018).

The ABDG method was used to delimit species using the COI gene. We used the online analysis platform (https://bioinfo.mnhn.fr/abi/public/abgd/abgdweb.html) and input data in the form FASTA file and the data was analyzed according to the site’s default mode using model Kimura 2-P. The output of the analysis is in Table S4.

*Cytogenetic analysis*

Mitotic chromosomes were obtained from cephalic kidney cells (Bertollo et al., 1978). For the detection of nucleolus organizer regions (NORs), the Ag-NOR technique (Howell and Black, 1980) was used, and constitutive heterochromatin was detected by the C-banding technique (Sumner, 1972). 

*Cartography*

Maps were made using QGIS v. 3.10.7. The shapefiles containing geographic data (elevation, hydrography, and country limits) were obtained from DIVA-GIS92, in the link https://www.diva-gis.org/gdata (Hijmans *et al*., 2004). The included coordinates were obtained from the present study, from data available on GenBank, and type localities according to appropriate references available on FishBase (Froese and Pauly, 2022).

## Results

### Molecular analysis

**Figure 2 -:**
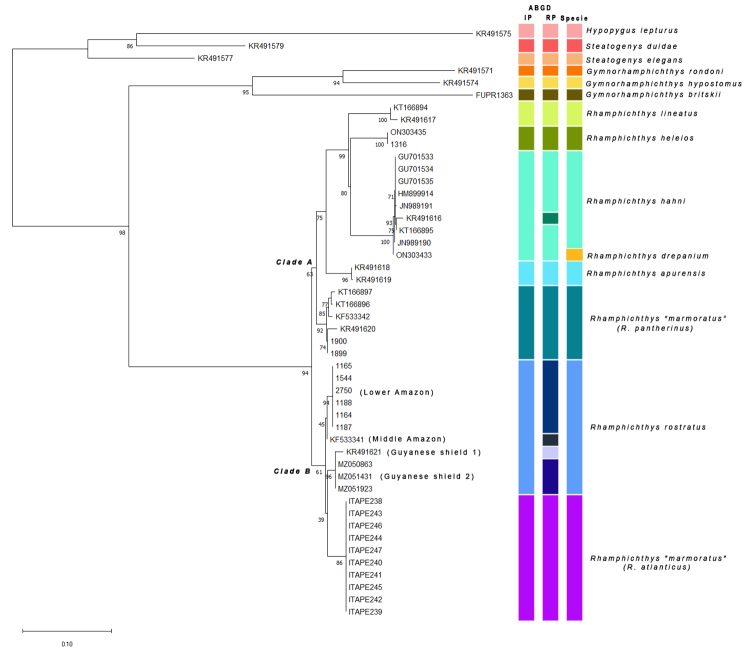
Maximum likelihood phylogenetic tree for *Rhamphichthys* samples, and outgroups, support values as percentages based on 1000 bootstrap pseudoreplicates. Clades A and B, blocks indicate species delimited by ABGD initial (IF) and recursive (RF) methods and unified morphological classification after reevaluation of the available literature.

Accumulating sequences available in databases such as GenBank, BOLD and newly produced in the present study, we evaluated a total of 42 COI gene sequences for species of the genus *Rhamphichthys*. The mitochondrial COI data showed that the smallest interspecific genetic distance was observed between *R. drepanium* and *R. hahni* (0.16%) while the highest was observed between *R. rostratus* (Guyana Shield) and *R. hahni* (9.9%) (Table S3). The genus *Rhamphichthys* was monophyletic to the other genera of the Rhamphichthyidae family according to the ML tree (Figure 2). The formation of two large groups of species with 94% bootstrap support was observed. The first clade (A) includes the species *R. lineatus*, *R. heleios*, *R. hahni*, *R. drepanium*, *R. apurensis* and *R. pantherinus*. The second clade (B) includes *R. rostratus* and *R. atlanticus*.

According to the results obtained by the ABDG species delimitation, a total of 7 to 11 species (based on either the initial or recursive partitions respectively) can be recovered considering intraspecific divergences (P) of 0.00167 to 0.0046. Using the initial partition as a reference, we have *R. lineatus, R. heleios,* (*R. hahni+R. drepanium*)*, R. apurensis*, and *R. pantherinus* in clade A, and *R. rostratus* and *R. atlanticus* for clade B, while in the recursive partition 11 species are recovered due to subdivisions of *R. hahni* and *R. rostratus* (Figure 2). The species *R. drepanium* remained included in *R. hahni* in all initial partitions with P ranging from >0.001 to 0.0215, only separating from *R. hahni* when haplotypes are identified as unique lineages (P of 0.001 in the recursive partition, see Table S4).

### Cytogenetic analysis

**Figure 3 -:**
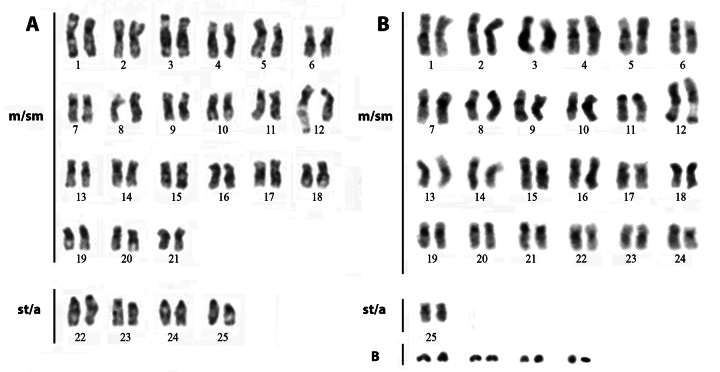
A) Karyotype of the species *Rhamphichthys pantherinus* with 2n = 50 (FC= 42m/sm+8st/y). B) Karyotype of *R. rostratus* with 2n = 50+8 B (FC= 48m/sm+2st/a+8B). Karyotypes demonstrate differentiation based on the distribution of constitutive heterochromatin through C-banding and highlighting of the NOR pair. Pair 12 appears very long because the NOR is located on the long arm, and in both metaphases this region is decondensed.

Samples identified as *Rhamphichthys pantherinus* presented 2n = 50/FN= 92 for samples from the Caripetuba River. The karyotypic formula observed was 42m/sm+8st/a with most chromosomes presenting pericentromeric heterochromatin (Figure 3 A ), but pairs with heterochromatic chromosomal arms were also observed, such as the long arm of pair 3 and the short arm of pair 11. The nucleolus organizer region (NOR) was found on pair 12 (highlighted in Figure 3 A).

Samples identified as *Rhamphichthys rostratus* presented 2n = 50/FN= 98 and the presence of B chromosomes in a variable number (5 to 10) was observed, according to the locality. The karyotypic formula observed was 48m/sm+2st/a + 5-10 Bs, where the sample from the Guamá River (Belém) presented 5-6 Bs (mode 6), from the Anequara and Caripetuba rivers 5-8 Bs (mode 8), and Arienga 6-10 Bs (mode 10) as detailed in Table 1. Most chromosomes show pericentromeric heterochromatin (Figure 3 B ), but pairs with heterochromatic chromosomal arms were also observed, such as the long arm of pair 3 and the short arm of pair 18. The nucleolus organizer region (NOR) was found on pair 12 (highlighted in Figure 3 B ).

## Discussion

DNA barcoding in fish, based on the sequencing of a segment of the Cytochrome C Oxidase type I (COI) gene, has proven to be a significant and accurate tool for species identification, authentication, and phylogenetic analysis (Bhattacharya et al., 2016). From the ML tree produced, we observe numerous inconsistencies with the allocation of the species names *R. marmoratus* (currently treated as a synonym of *R. pantherinus* Castelnau 1855) and *R. rostratus* to sequences from public databases that fall in various clades in our tree. Based on this, we reinforce the benefits of using DNA barcoding in facilitating the identification of species, highlighting the expansion of species already recognized or even signaling species previously neglected and allowing identifications where traditional methods cannot be applied (Ward et al., 2009). 

Despite a troubled taxonomic history (Triques, 1994, 1999), the genus *Rhamphichthys* proved to be monophyletic in our analyses based on the mitochondrial COI gene. However, we can observe the formation of two large clades. Clade A showed no problems regarding its species and included *R. lineatus*, *R. heleios*, *R. hahni*, *R. drepanium*, *R. apurensis*, and *R. pantherinus*. In this clade, we highlight the recently described species *R. heleios* (Carvalho and Albert, 2015) which proved to be valid and closely related to *R. lineatus*, *R. hahni,* and *R. drepanium*. Its karyotype was also identical to that described for *R. hahni* (2n = 50/FN= 94 KF = 44m/sm+6st/a) (Mendes et al., 2012), which can reinforce this proximity relationship. It is important to highlight in the grouping of these four species the low genetic divergence present between *R. drepanium* (Figure 1 A , Locality 14) and *R. hahni* (Figure 1 B , Locality 25 and 26) of less than 1% (0.16%) while about the other related species, it was greater than 6.5%. This low genetic divergence also appears in the ABGD analyses where *R. drepanium* is not recovered as a distinctly delimited species (Figure 2). This reflects that there must have been a recent separation between these two morphologically distinct species. Thus, we may be facing a river capture effect, a process that is important for the diversification of many Amazonian species (Albert *et al*., 2006; Tagliacollo et al., 2016). Dagosta and de Pinna (2017) argue that basins adjacent to the Amazon, such as Paraná-Paraguay, Essequibo, and Orinoco, for example, record many taxa with the Amazon basin and even species whose closest relatives are from the Amazon. In some cases, the separation between the Amazon and adjacent basins is incomplete and still allows for ichthyofaunistic exchange. Further investigation of these two species is needed to understand this probable dispersal event between the La Plata and Amazon basins and better describe their geographic distributions.

In clade B, according to the initial partition in ABDG, we observe the presence of the species *R. rostratus* and *R. atlanticus*. However, the recursive partition with P between 0.00167 to 0.0046 suggests the separation of four potential species present in *R. rostratus* here called *R. rostratus* Lower Amazon (Figure 1, Localities 1, 2 and 3), *R. rostratus* Middle Amazon (Figure 1, Locality 16), *R. rostratus* Guiana Shield 1 (Figura 1, Locality 5 - approximate) and Guiana Shield 2 (Figure 1 Localities 5, 6 and 8, respectively) according to the places of origin. Considering the phylogeny in Figure 2, we also infer that *R. rostratus* contains two main independent lineages, one occurring in the Northeast Basin of South America that is associated with the type locality of *R. rostratus* in Surinam (Figure 1 B , Locality 7) and another in the Amazon and Tocantins Basins (Figure 1 B , Locality 1, 2, 3 and 16). However, more sampling is required to determine the distinction of sublineages in each of these main lineages. *Rhamphichthys rostratus* is known to present similarities in morphology, color patterns, and habitat use (Reis et al., 2003) which makes identification difficult and consequently has contributed to an underestimation of the real species richness present in the genus overall. Thus, we emphasize the need for a more detailed view and deeper studies of the representatives of clade B to elucidate the possible hidden diversity present.

In a recent publication, Carvalho and Albert (2023) consider the species *Rhamphichthys apurensis*, *R. drepanium*, *R. hahni*, *R. heleios*, *R. lineatus*, *R. pantherinus*, and *R. rostratus* to be valid. Our data support these taxa as valid species. However, those authors propose that *R. atlanticus* and *R. longior* are junior synonyms of *R. pantherinus*. Our data do not agree with this proposition, at least for *R. atlanticus*, since *R. longior* was not analyzed in the present work. The COI analysis demonstrated a 6% difference between *R. pantherinus* and *R. atlanticus*, each of these taxa being in a different clade (Figure 2), while the difference between *R. atlanticus* and *R. rostratus*, from the same clade B, was 3%, and within *R. atlanticus* the distance was less than 1% (Table S3). The ABDG species delimitation analysis also recovers *R. atlanticus* as a valid species.

Despite having the same 2n=50, *R. rostratus* and *R. pantherinus* differ significantly in their karyotypes both in chromosomal morphology and in the presence of extra B chromosomes allowing clear differentiation of these sympatric species from their unique karyotypes. The data presented here are also different from those described for these species (Silva et al., 2013) in which 2n = 50/FN= 92 KF = 42m/sm+8st/a was associated with *R. rostratus* and 2n = 50/FN = 94 kF = 44m/sm+6st/a with *R. pantherinus* (cited as *R. marmoratus*)*.*

In our analyses based on the mitochondrial COI gene, we included specimens previously analyzed (Silva et al., 2013) (see Table S2) and we realized that there is a need to review the previously described information. Our data indicate that the karyotypes previously described (Silva *et al*., 2013) should be respectively associated with the species *R. pantherinus* (2n = 50/FN= 92 KF = 42m/sm+8st/a) and *R. heleios* (2n = 50/FN= 94 KF = 44m/sm+6st/a). A reason that may be responsible for the mistaken allocation of karyotypes was the lack of awareness of the existence of the species *R. heleios* which was only later described (Carvalho and Albert, 2015) in addition, the species of the genus have great morphological similarities (Reis et al., 2003).

For the three karyotyped species of *Rhamphichthys* of clade A, we observed the presence of two very similar karyotypes 2n = 50/FN= 92 KF = 42m/sm+8st/a for *R. pantherinus* and 50/FN= 94 KF = 44m/sm+6st/a associated with *R. heleios* and *R. hahni* while we can observe great differences about the karyotype present in *R. rostratus* Lower Amazon of clade B 2n = 50/FN= 98 KF = 48m/sm+2st/a+(5-10)Bs. These data demonstrate that, unlike the Hypopomidae family, which presents diploid number variations from 2n = 36 to 48 (de Jesus et al., 2016; Batista et al., 2017; Cardoso et al., 2018), the Rhamphichthyidae family presents conservation of the diploid number 2n=50 for all analyzed species. However, we can observe significant differences regarding the chromosomal macrostructure (Almeida-Toledo, 1978; Cardoso *et al*., 2011; Mendes et al., 2012; Silva et al., 2013).

From classical cytogenetics, we can infer that pericentric inversion events differentiate the karyotypes. However, as demonstrated through comparative studies of cytogenetic mapping in the genus *Gymnotus* (Nagamachi et al., 2010, 2013), species with almost identical karyotypes by classical cytogenetics had undergone a considerable amount of hidden karyotypic differences caused by rearrangements, being quite different. Thus, although the karyotypes of *Rhamphichthys* studied here are similar in a more superficial analysis, they may present a considerable number of hidden differences. Furthermore, we performed the first description of extranumerary B chromosomes in Rhamphichthyoidea associated with species *R. rostratus* Lower Amazon 2n = 50/FN= 98 KF = 48m/sm+2st/a+(5-10 Bs). The presence of supernumerary B chromosomes can be an autapomorphy of this species or even a synapomorphy of the B clade. Thus, we emphasize the need to carry out cytogenetic studies on the other representatives of this clade.

## Supplementary material

The following online material is available for this article:

Table S1 - Comparison of morphometric data presented by specimens of the genus Rhamphichthys collected sympatrically in the Caripetuba River, Abaetetuba - Pará.

Table S2 - Voucher specimens used on the molecular analyses (ML+ABGD).

Table S3 - P estimated distance for the COI gene calculated from the K2P model.

Table S4 - Results of the Automatic Barcode Gap Discovery (ABGD) analysis.

## Data Availability 

 The full dataset supporting the findings of this study is available upon request to the corresponding author.
