# Supplementary material for: Molecular and cytogenetic data for Rhamphichthys Müller and Troschel, 1848 (Rhamphichthyidae - Gymnotiformes): Evidence of chromosomal differentiation in R. heleios, R. hahni, R. rostratus and R. pantherinus
Source: Genet Mol Biol. 2026 Aug 3;49(Suppl 4):e20250092. doi: 10.1590/1678-4685-GMB-2025-0092 (PMC13450659; doi:10.1590/1678-4685-GMB-2025-0092)
Supplement: Supplementary file 1 [file 1415-4757-GMB-49-s4-e20250092-s1.pdf]

Genetics and Molecular Biology
https://doi.org/10.1590/1678-4685-GMB-2025-0092

Supplementary Material to "Molecular and cytogenetic data for *Rhamphichthys* Müller and Troschel, 1848 (Rhamphichthyidae - Gymnotiformes): Evidence of chromosomal differentiation in *R. heleios, R. hahni, R. rostratus* and *R. pantherinus*"

Table S1 - Comparison of morphometric data presented by specimens of the genus *Rhamphichthys*, collected sympatrically in the Caripetuba River, Abaetetuba – Pará.

Species	*R. pantherinus*		*R. rostratus*			
Karyotype Formulas	No B chromosome 42m/sm+8st/a		With B chromosome 48m/sm+2st/a + (5-10 B)			
copies	1899 (Sex not identified)	1900 (Female)	1187 (Eyes)	1188 (Female)	1544 (Eyes)	2750 (Eyes)
SL	399.2	356.61	458.12	516.24	405,78	481.68
AFL	361.62	326.29	399.62	468.84	355.30	434.90
PAFL	37.58	30.32	58.50	47.40	50.48	46.78
BD	44.66	28.70	43.82	42.18	39.34	45.52
PFL	22.30	17.26	23.08	20.0	19.80	20.78
HL	57.08	46.30	73.46	75.10	70.58	72.36
CFL	70.98	66.92	137.34	126.70	-	119.52
CFD	10.20	5.82	11.22	10.70	7.86	9.46
IOD	6.26	4.76	6.68	7.72	6.40	6.38
SL	28.94	23.46	45.10	43.02	43.72	43.0
POL	28.30	21.38	29.12	29.58	24.90	26.50
ED	4.12	2.40	3.32	3.48	3.28	3.32
DPN						
BO	12.14	6.32	9.86	9.08	8.74	9.38
Anal Rays	508	497	752	748	698	729
AFR	410	420	380	-	-	-
PFR	19¿	19	14/15/16			
pectoral rays	26	28	30	36	42	32
