# Supplementary material for: Molecular and cytogenetic data for Rhamphichthys Müller and Troschel, 1848 (Rhamphichthyidae - Gymnotiformes): Evidence of chromosomal differentiation in R. heleios, R. hahni, R. rostratus and R. pantherinus
Source: Genet Mol Biol. 2026 Aug 3;49(Suppl 4):e20250092. doi: 10.1590/1678-4685-GMB-2025-0092 (PMC13450659; doi:10.1590/1678-4685-GMB-2025-0092)
Supplement: Supplementary file 2 [file 1415-4757-GMB-49-s4-e20250092-s2.pdf]

Genetics and Molecular Biology
https://doi.org/10.1590/1678-4685-GMB-2025-0092

Supplementary Material to "Molecular and cytogenetic data for *Rhamphichthys* Müller and Troschel, 1848 (Rhamphichthyidae - Gymnotiformes): Evidence of chromosomal differentiation in *R. heleios, R. hahni, R. rostratus* and *R. pantherinus*"

Table S2 - Voucher specimens used on the molecular analyses (ML+ABGD). Names in quotation marks corresponding to those found in the consulted databases and names in parentheses corresponding to those identified in the present study.

Species	**Voucher/GenBank Acess**	**Coordinate**	**Reference**
Hypopygus lepturus	KR491575	unknown	Tagliacollo et al., [12]
Steatogenys duidae	KR491579	unknown	Tagliacollo et al., [12]
Steatogenys elegans	KR491577	3°45'51.33"S/ 73°15'1.90"O (approximate)	Tagliacollo et al., [12]
Gymnorhamphichthys rondoni	KR491571	4°40'8.46"N/ 66°33'35.78"O (approximate)	Tagliacollo et al., [12]
Gymnorhamphichthys hypostomus	KR491574	unknown	Tagliacollo et al., [12]
Gymnorhamphichthys britskii	FUPR1363	21°53'3.12"S/ 53°45'12.96"O	Pereira et al., [22]
Rhamphichthys lineatus	KT166894	3°38'34.00"S/ 73°12'50.00"O	Carvalho and Albert [3]
Rhamphichthys lineatus	KR491617	3°38'34.00"S/ 73°12'50.00"O	Carvalho and Albert [3]
Rhamphichthys heleios	ON303435	2°11'24.00"S/ 54°51'0.00"O	Janzen et al., [21]
Rhamphichthys heleios	1316	3° 7'32.50"S/ 64°46'47.30"O	Present Study
Rhamphichthys hahni	GU701534	22° 2'37.68"S/ 53°43'38.64"O	Pereira et al., [22]
Rhamphichthys hahni	GU701535	22° 2'37.68"S/ 53°43'38.64"O	Pereira et al., [22]
Rhamphichthys hahni	HM899914	22° 2'37.68"S/ 53°43'38.64"O	Pereira et al., [22]
Rhamphichthys hahni	JN989190	22°43'19.56"S/ 53°17'11.40"O	Pereira et al., [22]
Rhamphichthys hahni	JN989191	22° 2'37.68"S/ 53°43'38.64"O	Pereira et al., [22]
Rhamphichthys hahni	KR491616	22° 2'37.68"S/ 53°43'38.64"O	Pereira et al., [22]

Species	**Voucher/GenBank Acess**	**Coordinate**	**Reference**
Rhamphichthys hahni	KT166895	22° 2'37.68"S/ 53°43'38.64"O	Pereira et al., [22]
Rhamphichthys drepanium	ON303433	2°11'24.00"S/ 54°51'0.00"O	Janzen et al., [21]
Rhamphichthys apurensis	KR491618	7°36'0.00"N/ 66°12'0.00"O	Tagliacollo et al., [12]
Rhamphichthys apurensis	KR491619	7°36'0.00"N/ 66°12'0.00"O	Tagliacollo et al., [12]
Rhamphichthys rostratus	1165	1°28'33.88"S/ 48°27'8.73"O	Present Study
Rhamphichthys rostratus	1187	1°40'42.60"S/ 49° 0'16.60"O	Present Study
Rhamphichthys rostratus	1188	1°40'42.60"S/ 49° 0'16.60"O	Present Study
Rhamphichthys rostratus	1544	1°34'25.36"S/ 48°45'55.21"O	Present Study
Rhamphichthys rostratus	2750	1°34'25.36"S/ 48°45'55.21"O	Present Study
Rhamphichthys rostratus	1164	1°28'33.88"S/ 48°27'8.73"O	Present Study
Rhamphichthys rostratus	KF533341	2°46'32.81"S/ 60°44'35.52"O	Maldonado-Ocampo et al., [23]
Rhamphichthys rostratus	KR491621	3°48'21.96"N/ 54° 8'55.32"O (approximate)	Tagliacollo et al., [12]
Rhamphichthys rostratus	MZ050863	3°21'57.60"N/ 55°25'55.56"O	Papa et al., [24]
Rhamphichthys rostratus	MZ051431	3°40'2.28"N/ 53°57'40.68"O	Papa et al., [24]
Rhamphichthys rostratus	MZ051923	3°40'2.28"N/ 53°57'40.68"O	Papa et al., [24]
Rhamphichthys "marmoratus" (R. atlanticus)	ITAPE238	2°53'60.00"S/ 44°12'0.00"O	Birindelli, J.L (BOLD:ADC5116)
Rhamphichthys "marmoratus" (R. atlanticus)	ITAPE239	4°54'0.00"S/ 43°17'60.00"O	Birindelli, J.L (BOLD:ADC5116)
Rhamphichthys "marmoratus" (R. atlanticus)	ITAPE240	4°54'0.00"S/ 43°17'60.00"O	Birindelli, J.L (BOLD:ADC5116)
Rhamphichthys "marmoratus" (R. atlanticus)	ITAPE241	4°54'0.00"S/ 43°17'60.00"O	Birindelli, J.L (BOLD:ADC5116)
Rhamphichthys "marmoratus" (R. atlanticus)	ITAPE242	4°54'0.00"S/ 43°17'60.00"O	Birindelli, J.L (BOLD:ADC5116)
Rhamphichthys "marmoratus" (R. atlanticus)	ITAPE243	4°54'0.00"S/ 43°17'60.00"O	Birindelli, J.L (BOLD:ADC5116)
Rhamphichthys "marmoratus" (R. atlanticus)	ITAPE244	4°54'0.00"S/ 43°17'60.00"O	Birindelli, J.L (BOLD:ADC5116)
Rhamphichthys "marmoratus" (R. atlanticus)	ITAPE245	4°54'0.00"S/ 43°17'60.00"O	Birindelli, J.L (BOLD:ADC5116)
Rhamphichthys "marmoratus" (R. atlanticus)	ITAPE246	4°54'0.00"S/ 43°17'60.00"O	Birindelli, J.L (BOLD:ADC5116)

Species	**Voucher/GenBank Acess**	**Coordinate**	**Reference**
Rhamphichthys "marmoratus" (R. pantherinus)	KR491620	unknown	Tagliacollo et al., [12]
Rhamphichthys "rostratus (R. pantherinus)	KF533342	2°55'33.59"S/ 60°36'4.01"O	Maldonado-Ocampo et al. [23]
Rhamphichthys pantherinus	1899	1°37'23,49"S/48°55'33"O	Present Study
Rhamphichthys pantherinus	1900	1°37'23,49"S/48°55'33"O	Present Study
Rhamphichthys "marmoratus" (R. pantherinus)	KT166896	unknown	Carvalho,T.P. and Albert,J.S.
Rhamphichthys "marmoratus" (R. pantherinus)	KT166897	unknown	Carvalho,T.P. and Albert,J.S.
