# Supplementary material for: Molecular and cytogenetic data for Rhamphichthys Müller and Troschel, 1848 (Rhamphichthyidae - Gymnotiformes): Evidence of chromosomal differentiation in R. heleios, R. hahni, R. rostratus and R. pantherinus
Source: Genet Mol Biol. 2026 Aug 3;49(Suppl 4):e20250092. doi: 10.1590/1678-4685-GMB-2025-0092 (PMC13450659; doi:10.1590/1678-4685-GMB-2025-0092)
Supplement: Supplementary file 3 [file 1415-4757-GMB-49-s4-e20250092-s3.pdf]

Genetics and Molecular Biology
https://doi.org/10.1590/1678-4685-GMB-2025-0092

Supplementary Material to "Molecular and cytogenetic data for *Rhamphichthys* Müller and Troschel, 1848 (Rhamphichthyidae - Gymnotiformes): Evidence of chromosomal differentiation in *R. heleios, R. hahni, R. rostratus* and *R. pantherinus*"

Table S3 - P estimated distance for the COI gene calculated from the K2P model.

	Specie	1	2	3	4	5	6	7	8	9	10	11	12	13	14	15	16	17	18	19	20	21	22	23	24	25	26	27	28	29	30	31	32	33	34	35	36	37	38	39	40	41	42	43	44	45	46	47	48
1	KR491575 *Hypopygus lepturus*																																																
2	KR491579 *Steatogenys duidae*	0,18																																															
3	KR491577 *Steatogenys elegans*	0,16	0,13																																														
4	KR491571 *Gymnorhamphichthys rondoni*	0,19	0,20	0,19																																													
5	KR491574 *Gymnorhamphichthys hypostomus*	0,19	0,18	0,17	0,12																																												
6	FUPR1363 *Gymnorhamphichthys britskii*	0,19	0,18	0,17	0,17	0,17																																											
7	KT166894 *Rhamphichthys lineatus*	0,19	0,18	0,18	0,19	0,19	0,18																																										
8	KR491617 *Rhamphichthys lineatus*	0,18	0,18	0,18	0,18	0,18	0,19	0,01																																									
9	ON303435 *Rhamphichthys heleios*	0,19	0,19	0,21	0,19	0,19	0,19	0,07	0,08																																								
10	1316 *Rhamphichthys heleios*	0,19	0,19	0,21	0,18	0,19	0,19	0,07	0,08	0,00																																							
11	GU701533 *Rhamphichthys hahni*	0,20	0,19	0,20	0,19	0,18	0,18	0,07	0,08	0,07	0,07																																						
12	GU701534 *Rhamphichthys hahni*	0,20	0,19	0,20	0,19	0,18	0,18	0,07	0,08	0,07	0,07	0,00																																					
13	GU701535 *Rhamphichthys hahni*	0,20	0,19	0,20	0,19	0,18	0,18	0,07	0,08	0,07	0,07	0,00	0,00																																				
14	HM899914 *Rhamphichthys hahni*	0,20	0,19	0,20	0,19	0,18	0,18	0,07	0,08	0,07	0,07	0,00	0,00	0,00																																			
15	JN989190 *Rhamphichthys hahni*	0,20	0,19	0,20	0,19	0,18	0,18	0,07	0,08	0,07	0,07	0,00	0,00	0,00	0,00																																		
16	JN989191 *Rhamphichthys hahni*	0,21	0,19	0,20	0,19	0,19	0,19	0,07	0,08	0,07	0,07	0,00	0,00	0,00	0,00	0,00																																	
17	KR491616 *Rhamphichthys hahni*	0,19	0,18	0,19	0,18	0,18	0,19	0,08	0,07	0,08	0,07	0,01	0,01	0,01	0,01	0,01	0,01																																
18	KT166895 *Rhamphichthys hahni*	0,20	0,19	0,20	0,19	0,18	0,19	0,07	0,08	0,07	0,07	0,00	0,00	0,00	0,00	0,00	0,00	0,01																															
19	ON303433 *Rhamphichthys drepanium*	0,20	0,19	0,20	0,19	0,18	0,18	0,07	0,08	0,07	0,07	0,00	0,00	0,00	0,00	0,00	0,00	0,01	0,00																														
20	KR491618 *Rhamphichthys apurensis*	0,19	0,18	0,19	0,16	0,18	0,17	0,07	0,07	0,08	0,08	0,08	0,08	0,08	0,08	0,08	0,08	0,07	0,08	0,08																													
21	KR491619 *Rhamphichthys apurensis*	0,19	0,18	0,19	0,16	0,18	0,17	0,08	0,07	0,08	0,08	0,08	0,08	0,08	0,08	0,08	0,08	0,07	0,08	0,08	0,00																												
22	1165 *Rhamphichthys rostratus*	0,19	0,18	0,19	0,17	0,18	0,19	0,08	0,09	0,08	0,08	0,08	0,08	0,08	0,08	0,08	0,08	0,09	0,08	0,08	0,05	0,05																											

	Specie	1	2	3	4	5	6	7	8	9	10	11	12	13	14	15	16	17	18	19	20	21	22	23	24	25	26	27	28	29	30	31	32	33	34	35	36	37	38	39	40	41	42	43	44	45	46	47	48
23	1187 *Rhamphichthys rostratus*	0,19	0,18	0,19	0,17	0,18	0,19	0,08	0,09	0,08	0,08	0,08	0,08	0,08	0,08	0,08	0,08	0,09	0,08	0,08	0,05	0,05	0,00																										
24	1188 *Rhamphichthys rostratus*	0,19	0,18	0,19	0,17	0,18	0,19	0,08	0,09	0,08	0,08	0,08	0,08	0,08	0,08	0,08	0,08	0,09	0,08	0,08	0,05	0,05	0,00	0,00																									
25	1544 *Rhamphichthys rostratus*	0,19	0,18	0,19	0,17	0,18	0,19	0,08	0,09	0,08	0,08	0,08	0,08	0,08	0,08	0,08	0,08	0,09	0,08	0,08	0,05	0,05	0,00	0,00	0,00																								
26	2750 *Rhamphichthys rostratus*	0,19	0,18	0,19	0,17	0,18	0,19	0,08	0,09	0,08	0,08	0,08	0,08	0,08	0,08	0,08	0,08	0,09	0,08	0,08	0,05	0,05	0,00	0,00	0,00	0,00																							
27	1164 *Rhamphichthys rostratus*	0,19	0,18	0,19	0,17	0,18	0,19	0,08	0,09	0,08	0,08	0,08	0,08	0,08	0,08	0,08	0,08	0,09	0,08	0,08	0,05	0,05	0,00	0,00	0,00	0,00	0,00																						
28	KF533341 *Rhamphichthys rostratus*	0,19	0,18	0,19	0,17	0,17	0,18	0,08	0,09	0,08	0,08	0,08	0,08	0,08	0,08	0,08	0,08	0,09	0,08	0,08	0,05	0,05	0,01	0,01	0,01	0,01	0,01	0,01																					
29	KR491621 *Rhamphichthys rostratus*	0,18	0,18	0,18	0,17	0,16	0,18	0,09	0,09	0,09	0,09	0,09	0,09	0,09	0,10	0,10	0,10	0,09	0,09	0,09	0,05	0,05	0,02	0,02	0,02	0,02	0,02	0,02	0,02																				
30	MZ050863 *Rhamphichthys rostratus*	0,19	0,18	0,19	0,17	0,17	0,18	0,09	0,09	0,08	0,08	0,09	0,09	0,09	0,09	0,09	0,09	0,09	0,09	0,09	0,06	0,06	0,02	0,02	0,02	0,02	0,02	0,02	0,01	0,01																			
31	MZ051431 *Rhamphichthys rostratus*	0,19	0,18	0,19	0,17	0,17	0,18	0,09	0,09	0,08	0,08	0,09	0,09	0,09	0,09	0,09	0,09	0,09	0,09	0,09	0,06	0,06	0,02	0,02	0,02	0,02	0,02	0,02	0,01	0,01	0,00																		
32	MZ051923 *Rhamphichthys rostratus*	0,19	0,18	0,19	0,17	0,17	0,18	0,09	0,09	0,08	0,08	0,09	0,09	0,09	0,09	0,09	0,09	0,09	0,09	0,09	0,06	0,06	0,02	0,02	0,02	0,02	0,02	0,02	0,01	0,01	0,00	0,00																	
33	ITAPE238 *Rhamphichthys atlanticus*	0,19	0,18	0,19	0,18	0,17	0,18	0,09	0,10	0,07	0,07	0,09	0,09	0,09	0,09	0,09	0,09	0,10	0,09	0,09	0,07	0,07	0,03	0,03	0,03	0,03	0,03	0,03	0,02	0,04	0,03	0,03	0,03																
34	ITAPE239 *Rhamphichthys atlanticus*	0,19	0,18	0,19	0,18	0,17	0,18	0,09	0,10	0,07	0,07	0,09	0,09	0,09	0,09	0,09	0,09	0,10	0,09	0,09	0,07	0,07	0,03	0,03	0,03	0,03	0,03	0,03	0,02	0,04	0,03	0,03	0,03	0,00															
35	ITAPE240 *Rhamphichthys atlanticus*	0,19	0,18	0,19	0,18	0,17	0,18	0,09	0,10	0,07	0,07	0,09	0,09	0,09	0,09	0,09	0,09	0,10	0,09	0,09	0,07	0,07	0,03	0,03	0,03	0,03	0,03	0,03	0,02	0,04	0,03	0,03	0,03	0,00	0,00														
36	ITAPE241 *Rhamphichthys atlanticus*	0,19	0,18	0,19	0,18	0,17	0,18	0,09	0,10	0,07	0,07	0,09	0,09	0,09	0,09	0,09	0,09	0,10	0,09	0,09	0,07	0,07	0,03	0,03	0,03	0,03	0,03	0,03	0,02	0,04	0,03	0,03	0,03	0,00	0,00	0,00													
37	ITAPE242 *Rhamphichthys atlanticus*	0,19	0,18	0,19	0,18	0,17	0,18	0,09	0,10	0,07	0,07	0,09	0,09	0,09	0,09	0,09	0,09	0,10	0,09	0,09	0,07	0,07	0,03	0,03	0,03	0,03	0,03	0,03	0,02	0,04	0,03	0,03	0,03	0,00	0,00	0,00	0,00												
38	ITAPE243 *Rhamphichthys atlanticus*	0,19	0,18	0,19	0,18	0,17	0,18	0,09	0,10	0,07	0,07	0,09	0,09	0,09	0,09	0,09	0,09	0,10	0,09	0,09	0,07	0,07	0,03	0,03	0,03	0,03	0,03	0,03	0,02	0,04	0,03	0,03	0,03	0,00	0,00	0,00	0,00	0,00											
39	ITAPE244 *Rhamphichthys atlanticus*	0,19	0,18	0,19	0,18	0,17	0,18	0,09	0,10	0,07	0,07	0,09	0,09	0,09	0,09	0,09	0,09	0,10	0,09	0,09	0,07	0,07	0,03	0,03	0,03	0,03	0,03	0,03	0,02	0,04	0,03	0,03	0,03	0,00	0,00	0,00	0,00	0,00	0,00										
40	ITAPE245 *Rhamphichthys atlanticus*	0,19	0,18	0,19	0,18	0,17	0,18	0,09	0,10	0,07	0,07	0,09	0,09	0,09	0,09	0,09	0,09	0,10	0,09	0,09	0,07	0,07	0,03	0,03	0,03	0,03	0,03	0,03	0,02	0,04	0,03	0,03	0,03	0,00	0,00	0,00	0,00	0,00	0,00	0,00									
41	ITAPE246 *Rhamphichthys atlanticus*	0,19	0,18	0,19	0,18	0,17	0,18	0,09	0,10	0,07	0,07	0,09	0,09	0,09	0,09	0,09	0,09	0,10	0,09	0,09	0,07	0,07	0,03	0,03	0,03	0,03	0,03	0,03	0,02	0,04	0,03	0,03	0,03	0,00	0,00	0,00	0,00	0,00	0,00	0,00	0,00								
42	ITAPE247 *Rhamphichthys atlanticus*	0,19	0,18	0,19	0,18	0,17	0,18	0,09	0,10	0,07	0,07	0,09	0,09	0,09	0,09	0,09	0,09	0,10	0,09	0,09	0,07	0,07	0,03	0,03	0,03	0,03	0,03	0,03	0,02	0,04	0,03	0,03	0,03	0,00	0,00	0,00	0,00	0,00	0,00	0,00	0,00	0,00							
43	KT166897 *Rhamphichthys pantherinus*	0,19	0,19	0,18	0,17	0,18	0,18	0,08	0,09	0,07	0,07	0,07	0,07	0,07	0,07	0,07	0,07	0,08	0,07	0,07	0,05	0,05	0,04	0,04	0,04	0,04	0,04	0,04	0,04	0,05	0,04	0,04	0,04	0,05	0,05	0,05	0,05	0,05	0,05	0,05	0,05	0,05	0,05						
44	KT166896 *Rhamphichthys pantherinus*	0,19	0,19	0,18	0,17	0,18	0,17	0,08	0,09	0,07	0,07	0,07	0,07	0,07	0,07	0,07	0,07	0,08	0,07	0,07	0,05	0,05	0,04	0,04	0,04	0,04	0,04	0,04	0,04	0,05	0,04	0,04	0,04	0,05	0,05	0,05	0,05	0,05	0,05	0,05	0,05	0,05	0,05	0,01					
45	KR491620 *Rhamphichthys pantherinus*	0,18	0,18	0,18	0,17	0,17	0,18	0,09	0,08	0,08	0,08	0,09	0,09	0,09	0,09	0,09	0,09	0,08	0,09	0,09	0,04	0,04	0,04	0,04	0,04	0,04	0,04	0,04	0,04	0,04	0,05	0,05	0,05	0,06	0,06	0,06	0,06	0,06	0,06	0,06	0,06	0,06	0,06	0,02	0,02				
46	KF533342 *Rhamphichthys pantherinus*	0,19	0,19	0,19	0,17	0,19	0,18	0,07	0,08	0,07	0,07	0,07	0,07	0,07	0,07	0,07	0,07	0,08	0,07	0,07	0,04	0,05	0,04	0,04	0,04	0,04	0,04	0,04	0,04	0,05	0,04	0,04	0,04	0,05	0,05	0,05	0,05	0,05	0,05	0,05	0,05	0,05	0,05	0,01	0,01	0,02			
47	1900 *Rhamphichthys pantherinus*	0,18	0,19	0,19	0,17	0,18	0,17	0,07	0,08	0,07	0,07	0,08	0,08	0,08	0,08	0,08	0,08	0,08	0,08	0,07	0,04	0,04	0,03	0,03	0,03	0,03	0,03	0,03	0,03	0,04	0,03	0,03	0,03	0,04	0,04	0,04	0,04	0,04	0,04	0,04	0,04	0,04	0,04	0,01	0,01	0,01	0,01		
48	1899 *Rhamphichthys pantherinus*	0,18	0,19	0,19	0,17	0,18	0,17	0,07	0,08	0,07	0,07	0,08	0,08	0,08	0,08	0,08	0,08	0,08	0,08	0,07	0,04	0,04	0,03	0,03	0,03	0,03	0,03	0,03	0,03	0,04	0,03	0,03	0,03	0,04	0,04	0,04	0,04	0,04	0,04	0,04	0,04	0,04	0,04	0,01	0,01	0,01	0,01	0,00	
