# Supplementary material for: Molecular and cytogenetic data for Rhamphichthys Müller and Troschel, 1848 (Rhamphichthyidae - Gymnotiformes): Evidence of chromosomal differentiation in R. heleios, R. hahni, R. rostratus and R. pantherinus
Source: Genet Mol Biol. 2026 Aug 3;49(Suppl 4):e20250092. doi: 10.1590/1678-4685-GMB-2025-0092 (PMC13450659; doi:10.1590/1678-4685-GMB-2025-0092)
Supplement: Supplementary file 4 [file 1415-4757-GMB-49-s4-e20250092-s4.pdf]

Genetics and Molecular Biology
https://doi.org/10.1590/1678-4685-GMB-2025-0092

Supplementary Material to "Molecular and cytogenetic data for *Rhamphichthys* Müller and Troschel, 1848 (Rhamphichthyidae - Gymnotiformes): Evidence of chromosomal differentiation in *R. heleios, R. hahni, R. rostratus* and *R. pantherinus*"

Table S4 - Results of the Automatic Barcode Gap Discovery (ABGD) analysis. X, relative gap width; Simple, p-distance; K2P, Kimura 2-parameter substitution model.

Sub. model	X	Partition	Prior intraspecific divergence (P)						
			0,001	0,00167	0,00278	0,0046	0,0077	0,0129	0,0215
K2P	1.5	Initial	13	13	13	13	13	13	13
	1.5	Recursive	19	17	17	17	12		
Simple	1.5	Initial	13	13	13	13	13	13	13
	1.5	Recursive	19	17	17	17			
